# Evaluation of a Four-Gene Panel for Hereditary Cancer Risk Assessment

**DOI:** 10.3390/genes13040682

**Published:** 2022-04-13

**Authors:** Angela Secondino, Flavio Starnone, Iolanda Veneruso, Maria Antonietta Di Tella, Serena Conato, Carmine De Angelis, Sabino De Placido, Valeria D’Argenio

**Affiliations:** 1 Department of Molecular Medicine and Medical Biotechnologies, Federico II University, Via Sergio Pansini 5, 80131 Napoli, Italy; secondino@ceinge.unina.it (A.S.); venerusoi@ceinge.unina.it (I.V.); 2 CEINGE-Biotecnologie Avanzate, Via G. Salvatore 486, 80145 Napoli, Italy; starnone@ceinge.unina.it (F.S.); ditella@ceinge.unina.it (M.A.D.T.); conato@ceinge.unina.it (S.C.); 3 Department of Clinical Medicine and Surgery, Federico II University, Via Sergio Pansini 5, 80131 Napoli, Italy; carmine.deangelis1@unina.it (C.D.A.); sabino.deplacido@unina.it (S.D.P.); 4 Department of Human Sciences and Quality of Life Promotion, San Raffaele Open University, Via di Val Cannuta 247, 00166 Roma, Italy

**Keywords:** breast cancer, ovarian cancer, *BRCA1*, *BRCA2*, *CHEK2*, *PALB2*, multigene panel testing, hereditary cancers, next generation sequencing

## Abstract

*BRCA1*/*2* are tumor suppressor genes involved in DNA double-strand break repair. They are the most penetrant genes for hereditary breast and ovarian cancers, but pathogenic variants in these two genes can be identified only in a fraction of hereditary cases. Following the diffusion of *BRCA* molecular testing and the availability of specific therapeutic strategies for the management of pathogenic variant carriers, the demand for the analysis of additional predisposing genetic factors has increased. Indeed, there is accumulating evidence regarding the role of other genes, including *CHEK2* and *PALB2.* Both of them are involved in the same molecular pathway as *BRCA* genes, with *CHEK2* being responsible for cell cycle stopping to allow the repair of DNA double-strand breaks and *PALB2* being able to interact with *BRCA1* and activate *BRCA2*. Thus, their role as additional hereditary cancer predisposing factors is intriguing. Accordingly, guidelines for hereditary cancer risk assessment have been updated to include the criteria for additional genes testing. In this context, we validated a commercially available kit allowing for the simultaneous analysis of *BRCA1*, *BRCA2*, *CHEK2* and *PALB2*. Forty-eight patients, already tested for *BRCA* mutational status, were re-analyzed in the present study. Results comparison showed that the tested method was able to correctly identify all the variants previously detected in the same patients. In particular, all single-nucleotide variants and small indels were correctly identified. Moreover, two copy number variants, included to assess the software’s performance in detecting this kind of gene alteration, were also detected. Even if copy number variant estimation still requires confirmation by a molecular technique to avoid false positive results, it is able to reduce the number of patients requiring multiplex ligation probe amplification analysis, positively impacting the test’s turnaround time. Finally, since the time and costs of the analysis are similar to those required just for *BRCA* genes, this strategy may be affordable for providing a more comprehensive test for hereditary cancer risk assessment.

## 1. Introduction

Breast cancer susceptibility genes 1 and 2 (*BRCA1* and *BRCA2*, OMIM #113705 and #600185, respectively) are the best known, most well established, and highly penetrant genes associated with increased risk of developing breast and ovarian cancers [1,2,3]. Since their discovery in the early 1990s [4], the role of *BRCA1* and *BRCA2* as tumor suppressor genes has been established due to their involvement in DNA double-strand break repair mechanisms [5]. Accordingly, several germline variants that are able to impair BRCA1/2 functions have been identified so far and have been related to an increased lifetime risk of developing cancers, particularly breast and ovarian cancers, within the framework of the so-called hereditary breast and ovarian cancer syndrome (HBOC) [2,3,6,7]. The identification of a predisposing germline variant in an affected patient supports his/her clinical and therapeutic management [8,9]. Moreover, it makes it possible to expand the analysis to the patient’s family members, offering the chance to identify other at-risk subjects. Based on these observations, specific guidelines have been released to regulate genetic test access [10,11,12], and these kinds of molecular investigations are currently widely performed [13]. This wide use of *BRCA* testing has also been greatly promoted by the availability of novel drugs with proven efficacy in the mutations’ carriers, i.e., poly(ADP-ribose) polymerase (PARP) inhibitors [14,15].

Despite this great interest, it is necessary to point out that pathogenic variants in *BRCA* genes are able to explain just a small fraction of hereditary cases, thus suggesting the increasingly emerging involvement of other predisposing genetic markers [16,17,18,19]. The diffusion of next-generation sequencing (NGS)-based approaches, as in the case of other genetically heterogeneous diseases [20,21], has greatly improved the study of the molecular basis of hereditary cancers. Indeed, not only does it provide for a fast, cost-effective and accurate *BRCA* gene analysis compared to traditional methodological approaches [22,23], but it also allows the identification of additional predisposing genes. In this context, several groups have assessed the utility of expanding the molecular test for the simultaneous analysis of a panel of genes in an attempt to identify pathogenic variants in moderate- or low-penetrance genes and discover new gene/disease associations [24,25,26,27,28]. Even if these studies are showing promising results, representing a basis for the advancement of knowledge and the design of novel tests, they may lack specific associations or functional verifications confirming the significance of their findings. Moreover, as in all the NGS-based analysis, they are risky for incidental findings and a potentially high number of variants of uncertain significance (VUSs), thus opening the way to ethical concerns and patient distress [29,30]. Taken together, these factors may limit the diffusion of such expanded genetic testing in a diagnostic context, especially if the provided results may be not actionable from a clinical point of view.

In this regard, the clinical actionability of multigene panels testing for HBOC assessment has been evaluated; indeed, the use of expanded panels is able to identify more at-risk patients with respect to *BRCA1/2* testing alone, and can provide useful information for both patient monitoring and familial testing, especially if the additional pathogenic variants are identified in the most common moderate-risk genes for breast and ovarian cancers, such as *CHEK2* and *PALB2* [31].

*CHEK2* (checkpoint kinase 2, OMIM #604373) codes for a protein involved in DNA repair mechanisms by regulating cell cycle stopping in the presence of a double-strand break [32]. *CHEK2* germline pathogenic variants have been associated with a lifetime risk of developing breast cancer of 37% and also to an increased risk of developing other kinds of cancer [33].

*PALB2* (partner and localizer of BRCA2, OMIM #610355) codes for a protein involved in the homologous recombination repair system. In particular, PALB2 interacts with the BRCA1/BARD1 complex and activates BRCA2, promoting the assembly of RAD51, thus allowing DNA repair [34]. *PALB2* is considered a high/moderate breast cancer risk gene, since germline pathogenic variants confer a lifetime risk of developing breast cancer of 53% [35]. 

Accordingly, specific recommendations for both *CHEK2* and *PALB2* testing and carrier monitoring have been included in the National Comprehensive Cancer Network (NCCN) guidelines for breast and/or ovarian cancer susceptibility evaluation; moreover, an association with limited strength of evidence has also been reported between *CHEK2* pathogenic variants and colon cancer risk and *PALB2* pathogenic variants and pancreatic cancer risk [12]. Indeed, it has to be underlined that, beyond breast and ovarian cancers, pathogenic variants in *BRCA1* and *BRCA2*, as well as in *CHEK2* and *PALB2,* have been associated with a higher risk of developing an increasing number of cancers. In particular, it has recently been reported that not only may *BRCA1/2* mutation frequency be higher than previous estimations in prostate cancer [36], but also that pathogenic variants in other genes involved in DNA double-strand break repair, like *CHEK2* and *PALB2,* may also play a role [37]. Similarly, data are accumulating regarding the incidence of germline mutations in these genes in pancreatic cancer patients [35,38]. Thus, the early identification of predisposing germline mutations in *BRCA1*, *BRCA2*, *CHEK2* and *PALB2* have the potential to positively impact the clinical management of these patients in view of an even more tailored medicine.

Based on the above, here we report the evaluation of a commercially available four-gene panel, including *BRCA1*, *BRCA2*, *CHEK2* and *PALB2*, to (i) assess the analytic features of the tested procedure; and (ii) verify the presence of additional pathogenic variants in *CHEK2* and *PALB2* genes. 

## 2. Materials and Methods

### 2.1. Patient Selection and DNA Sample Collection

**Figure 1:**
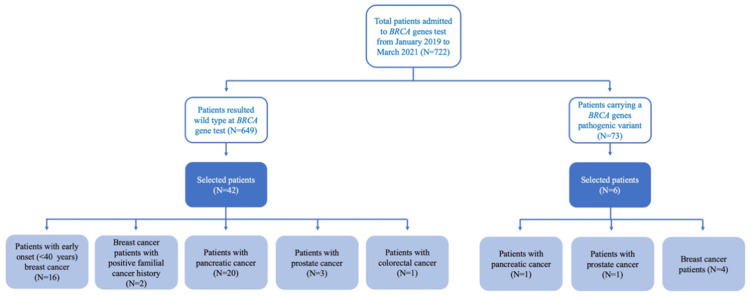
Strobe diagram representing the flow-chart of patient selection. In total, 48 samples were included in this study: 6 were carriers of a pathogenic variant in *BRCA1/2* and were included as analytic controls; 42 patients were determined as wild type after the diagnostic genetic test showing different clinical phenotypes, and were included to verify the presence of pathogenic, cancer-risk variants in the newly tested genes (i.e., *PALB2* and *CHEK2*).

The samples included in the present study were selected from among those of patients attending the CEINGE Biotecnologie Avanzate diagnostic laboratories for molecular analysis of *BRCA* genes. In particular, to ensure the availability of good-quality samples, we restricted the selection window to the period ranging from January 2019 to March 2021. During these 27 months, a total of 722 samples were analyzed, 73 (about 10%) being carriers of a pathogenic variant of the *BRCA1* or *BRCA2* genes, while the remaining 649 were wild type. A total of 48 samples were selected to be analyzed in the present study. In particular, to ensure the reliability of the tested methodological procedure in identifying different kinds of DNA variants, samples carrying an already identified pathogenic variant in *BRCA1/2* genes were included (N = 6). Moreover, to verify the contribution of *PALB2* and *CHEK2* in cancer predisposition, the remaining 42 samples were selected from among the patients resulted negative after the diagnostic test according to the following criteria: (i) patients with early onset (under 40 years of age) breast cancer (N = 16); (ii) patients with breast cancer and positive family history for breast and other kinds of cancer (N = 2); (iii) patients with pancreatic (N = 20), prostate (N = 3) or colorectal cancers (N = 1) (Figure 1).

Nineteen of these 48 samples were taken from male patients, 14 being affected by pancreatic cancer, 4 by prostate cancer and 1 by colorectal cancer. The remaining 29 samples were taken from female patients, of which 7 had pancreatic cancer and 22 breast cancer (16 with an age of cancer onset <40 years).

All the patients analyzed in this study gave their written informed consent to the anonymous use of their biological samples for research purposes.

### 2.2. DNA Library Preparation and Next-Generation Sequencing

Genomic DNA was extracted from a blood EDTA sample/patient using the Maxwell 16 instrument (Promega, Madison, WI, USA), quantified using the NanoDrop spectrophotometer instrument (Thermo Fisher Scientific Inc., Waltham, MA, USA), and verified for their integrity on the genomic screentape of the TapeStation (Agilent Technologies, Santa Clara, CA, USA).

Library preparation was carried out using the SureMASTR BRCA Screen with drMID for Illumina NGS systems protocol (Agilent Technologies, Santa Clara, CA, USA), according to the manufacturer’s instructions. Specifically, 50 ng of each DNA sample was PCR amplified to specifically enrich the *BRCA1*, *BRCA2*, *PALB2* and *CHEK2* target gene exons and their intronic flanking regions. Next, after a magnetic beads-based purification step using AMPure XP beads (Beckman Coulter, Brea, CA, USA), these amplicons were admitted to a second-step PCR, which was required for adding the universal adaptors for the following NGS reactions, and the specific sample indexes to unequivocally tag each sample. The obtained amplicon libraries were bead-purified and assessed for quality (TapeStation, Agilent Technologies, Santa Clara, CA, USA) and quantity (Qubit, dsDNA HS Assay, Life Technologies, Carlsbad, CA, USA). Thus, equimolar ratios of 24 different libraries were pooled for sequencing in a single sequencing run. Sequencing reactions were carried out using the flowcell V2 PE 2 × 150 and MiSeq instruments (Illumina, San Diego, CA, USA). The library pool was loaded at a final concentration of 8 pM with a 5% PhiX.

### 2.3. Sequence Data Analysis

The FASTQ files generated for each analyzed sample at the end of the sequencing reactions were used as input files for the downstream bioinformatic analysis. Specifically, the MASTR Reporter software (https://mr.agilent.com; accessed on 15 March 2022) was used for this purpose. This web-based application tool, specifically coupled with the library preparation reagents, allows easy and fast analysis of the sequence reads. Indeed, after the alignment of the reads against the reference target regions, a VCF file/sample is generated, which can be used to call any point variation and/or small ins/del identified in the analyzed sample. Moreover, the software integrates a pipeline for copy number variant (CNV) estimation based on: (i) the quantitative analysis of the obtained reads; and (ii) their normalization within the different amplicon of the same sample and between the different samples analyzed in the same sequencing run. In this way, for each sample, it is possible to obtain a list of variants for further analysis and the estimate of the CNV status for further evaluations. Variants’ significance was evaluated and assigned using dbSNP (https://www.ncbi.nlm.nih.gov/snp; accessed on 15 March 2022), ClinVar (https://www.ncbi.nlm.nih.gov/clinvar; accessed on 15 March 2022) and Varsome (https://varsome.com; accessed on 15 March 2022) databases.

### 2.4. Variant Validation

DNA variants, including single-nucleotide variants (SNPs), small ins/del, and CNVs, identified through the method tested in the current study in the *PALB2* and *CHEK2* genes, were further verified using a second independent molecular technique. Specifically, PCR-specific amplification followed by Sanger sequencing was performed for point variants and/or small ins/del. After amplicon verification on a 2% agarose gel, sequencing was carried out using an ABI 3100 capillary sequencer (Applied Biosystems Inc., Foster City, CA, USA). The obtained electropherograms were analyzed using the SeqMan tool (DNASTAR, Inc., Madison, WI, USA). For CNV validation, multiplex ligation probe amplification (MLPA) was carried out using gene-specific SALSA MLPA probe sets (MRC-Holland, Amsterdam, the Netherlands) and an ABI PRISM 3130 XL genetic analyzer (Applied Biosystems Inc., Foster City, CA, USA) for fragment separation. Finally, the Coffalyser software (MRC-Holland, Amsterdam, the Netherlands) was used for MLPA results analysis, according to the manufacturer’s instructions.

## 3. Results

The 48 samples included in the present study were analyzed as described in the Materials and Methods section. All the selected DNA samples were preliminarily checked for their integrity to avoid: (i) library preparation failure; or (ii) non-homogeneous amplification and representation of the genomic targets due to the use of degraded input samples. All the samples showed a DIN (DNA integrity number) ranging between 8.3 and 10, as assessed by the TapeStation using a genomic DNA screentape. Thus, all of them were used to obtain a DNA library for the downstream NGS analysis. Specifically, two sequencing runs (each one including 24 differently indexed libraries) were performed in total to ensure samples with a high sequencing coverage, as required for carrying out CNV estimation. An average of 6 Gb was obtained from each sequencing run with more than 90% of clusters passing filters and a Q30 of 84%, thus resulting in an average of 760 k reads/sample. These reads were analyzed using the MASTR Reporter software to highlight any differences with respect to the reference. The total number of variants identified in each gene is reported in Table 1, while the full list of identified SNPs and CNVs in each study subject is reported in Table 2 and Table 3, respectively

### 3.1. Assessment of Method Reliability in Variant Calling

The first aim of this study was to assess the analytical reliability of the tested procedure with respect to the currently used diagnostic procedure. As the *BRCA* genes had already been tested in all 48 samples for diagnostic purposes, we verified the consistency between the two methods. All 227 *BRCA* variants previously detected by the routine diagnostic procedure were also identified in the present analysis (Table 1, Table 2 and Table 3). Indeed, not only were the six pathogenic variants (including both single-nucleotide variants and CNVs) correctly identified, but the common benign variants present in each patient in these two genes were also correctly called (Table 2 and Table 3). This comparison ensures the reliability of the tested analytic procedure in correctly identify different kinds of DNA variants. As stated before, no false negative results were reported. However, the CNV estimation highlighted two additional possible deletions, one in *BRCA1* and the other in *BRCA2*, not reported by the previous analysis. MLPA evaluation of these variants did not confirm them, so we concluded that these represented false positive calling by the analysis software (Table 3).

### 3.2. Evaluation of Additional Variants Identified by the Tested Procedure

The next step was to verify the presence of additional pathogenic and clinically relevant variants in *CHEK2* and *PALB2* genes that may be associated with increased cancer risk in the analyzed samples. Interestingly, with respect to the highly polymorphic *BRCA* genes, no SNPs or small ins/del were detected at all in *CHEK2* and only 26 were identified in *PALB2* (Table 1). Among the latter, 25 were already-known variants, classified in the ClinVar database as benign variants, and one was classified as a variant of uncertain significance (VUS). Indeed, the c.3451C > T p.(Leu1151Phe) (rs786203462) in exon 13 of *PALB2* was identified in one of the analyzed patients and confirmed by Sanger sequencing (Table 2). This variant is currently classified as a VUS according to the ClinVar database. In particular, it replaces a leucine with a phenylalanine and, even if the substitution affects a highly conserved residue, the two amino acids have small physiochemical differences. Moreover, this variant has been identified with an allele frequency of 0.00000657 in the general population by the Genome Aggregation Database (gnomAD), but no functional studies have been performed to date to assess its pathogenicity and the prediction algorithms show inconclusive results on its potential effects (according to the Varsome database). To date, there are no published studies reporting this variant in association with hereditary cancer. However, it has to be noted that the above-mentioned variant falls within the PALB2 domain involved in the interaction with BRCA2 and RAD51, and is therefore of potential interest from a functional point of view. Accordingly, most of the already-known *PALB2* pathogenic variants fall within the *PALB2* C-terminus region, that is involved in the interaction with BRCA2.

Finally, CNV estimation highlighted a potential deletion involving exon 12 of *CHEK2* in one patient. MLPA was carried out to verify its presence, showing no alterations (Table 3). 

## 4. Discussion

Molecular testing for the identification of patients carrying cancer-predisposing mutations has become a routine practice due to the availability of laboratory procedures that enable the timely and cost-effective analysis of disease-related genes. In this context, *BRCA* gene testing represents a case-model. Indeed, not only do these genes have a strong association with HBOC risk, but the identification of pathogenic variants makes it possible to plan specific preventive and/or therapeutic strategies. As a consequence, the diffusion of this kind of test has greatly increased over the last decade [39]. On the other hand, since pathogenic variants in the *BRCA* genes explain only a portion of all hereditary cases, increasing evidence is accumulating regarding the role of other predisposing genes [24,25,26,27,28]. Among these, *CHEK2* and *PALB2*, two of the genes involved in the homologous recombination pathway, have been associated with an increased risk of breast and other cancers, and are included in the NCCN guidelines for hereditary cancer risk assessment [12]. Moreover, PARP inhibitors have also shown their efficacy in the presence of alterations affecting these two genes [15]. Thus, we tested the reliability of a four-gene panel allowing the simultaneous analysis of *BRCA1*, *BRCA2*, *CHEK2* and *PALB2*. 

In this evaluation study, we analyzed a total of 48 samples already tested for *BRCA* status. In this way, we were able to compare the analytic performance of the tested procedure and to assess whether all of the previously detected genes variants were correctly identified. It has to be noted that five CNVs in total were predicted following sequence read analysis. Two of them were already-known variants, a duplication of *BRCA1* exon 2 and a deletion involving *BRCA1* exons 16 and 17, which had already been detected by the diagnostic procedure and chosen as positive controls for assessing the ability of the tested method to correctly identify potential CNVs. The remaining three called CNVs were not confirmed by MLPA. Currently, there is a great interest in the development of bioinformatic strategies that, taking advantage of the high coverage allowed by NGS, can use it to estimate the presence of CNVs in the analyzed genes [40]. Indeed, this will make it possible to increase the diagnostic value of the analysis, since it is potentially able to detect a large spectrum of gene alterations. Based on our experience, we observed that, even though some CNVs were incorrectly called, no false negative results were reported. Thus, even if the predicted CNVs still require validation using a molecular technique, such as MPLA, NGS data analysis makes it possible to restrict this procedure to just a small number of cases, thus positively impacting molecular testing turnaround time. In this regard, since the costs and time of the analysis were similar to those required for the analysis of *BRCA* genes alone, this strategy may be an affordable alternative for detecting a high number of at-risk subjects. In our study group, we were not able to identify any pathogenic variants in the two additional genes. However, we analyzed just 42 *BRCA*-negative subjects; moreover, we included many pancreatic cancer patients based on *PALB2* association with this kind of cancer. Therefore, the estimation of the contribution of *CHEK2* and *PALB2* pathogenic variants in hereditary breast and ovarian cancer risk is not possible based on the data presented herein, and is beyond the scope of this study. Further studies including high numbers of patients have to be carried out to address this issue.

## 5. Conclusions

The evaluation study reported herein assesses the reliability of a four-gene panel for the simultaneous analysis of *BRCA1*, *BRCA2*, *CHEK2* and *PALB2.* Since the time and the costs of the tests are not impacted by the additional two genes with respect to classical *BRCA* evaluation, this strategy may be an effective alternative making it possible to increase the diagnostic sensitivity by identifying a higher number of at-risk patients.

## Figures and Tables

**Table 1:** Variants detected by the tested method in each of the analyzed genes.

Gene	Single-Nucleotide Variants and Ins/del	Copy Number Variants	Total
*BRCA1*	138	3	141
*BRCA2*	85	1	86
*CHEK2*	0	1	1
*PALB2*	26	0	26
Total	249	5	254

**Table 2:** Full list of single-nucleotide variants and small ins/del identified in each analyzed patient. Pathogenic variants are reported in bold.

Sample ID	Gender	Age at Diagnosis (y)	Kind of Cancer	Gene	Cdna *	Protein *	Reference SNP ID	Status	Clinvar Classification
P1	M	60	Pancreas	*BRCA1*	c.4900A > G	p.(Ser1634Gly)	rs1799966	Het	Benign
					c.3548A > G	p.(Lys1183Arg)	rs16942	Het	Benign
					c.3113A > G	p.(Glu1038Gly)	rs16941	Het	Benign
					c.2612C > T	p.(Pro871Leu)	rs799917	Het	Benign
					c.2596C > T	p.(Arg866Cys)	rs41286300	Het	Benign
					c.2077G > A	p.(Asp693Asn)	rs4986850	Het	Benign
					c.1067A > G	p.(Gln356Arg)	rs1799950	Het	Benign
				*BRCA2*	c.7397T > C	p.(Val2466Ala)	rs169547	Hom	Benign
				*PALB2*	c.2993G > A	p.(Gly998Glu)	rs45551636	Het	Benign
					c.2014G > C	p.(Glu672Gln)	rs45532440	Het	Benign
					c.1676A > G	p.(Gln559Arg)	rs152451	Het	Benign
P2	M	42	Pancreas	*BRCA1*	c.3119G > A	p.(Ser1040Asn)	rs4986852	Het	Benign
				*BRCA2*	c.7397T > C	p.(Val2466Ala)	rs169547	Hom	Benign
P3	M	66	Pancreas	*BRCA2*	c.3055C > G	p.(Leu1019Val)	rs55638633	Het	Benign
					c.7397T > C	p.(Val2466Ala)	rs169547	Hom	Benign
				*PALB2*	c.1676A > G	p.(Gln559Arg)	rs152451	Het	Benign
P4	M	69	Pancreas	*BRCA2*	**c.67+1G > A**	**p.(?)**	**rs81002796**	**Het**	**Pathogenic**
					c.1114A > C	p.(Asn372His)	rs144848	Het	Benign
					c.7397T > C	p.(Val2466Ala)	rs169547	Hom	Benign
				*PALB2*	c.2993G > A	p.(Gly998Glu)	rs45551636	Het	Benign
					c.2014G > C	p.(Glu672Gln)	rs45532440	Het	Benign
					c.1676A > G	p.(Gln559Arg)	rs152451	Het	Benign
P5	M	81	Colorectal	*BRCA1*	c.1067A > G	p.(Gln356Arg)	rs1799950	Hom	Benign
				*BRCA2*	c.1114A > C	p.(Asn372His)	rs144848	Het	Benign
					c.7397T > C	p.(Val2466Ala)	rs169547	Hom	Benign
P6	M	45	Prostate	*BRCA1*	c.4900A > G	p.(Ser1634Gly)	rs1799966	Het	Benign
					c.3548A > G	p.(Lys1183Arg)	rs16942	Het	Benign
					c.3113A > G	p.(Glu1038Gly)	rs16941	Het	Benign
					c.2612C > T	p.(Pro871Leu)	rs799917	Het	Benign
				*BRCA2*	c.7397T > C	p.(Val2466Ala)	rs169547	Hom	Benign
P7	M	65	Prostate	*BRCA1*	c.4900A > G	p.(Ser1634Gly)	rs1799966	Het	Benign
					c.3548A > G	p.(Lys1183Arg)	rs16942	Het	Benign
					c.3119G > A	p.(Ser1040Asn)	rs4986852	Het	Benign
					c.3113A > G	p.(Glu1038Gly)	rs16941	Het	Benign
					c.2612C > T	p.(Pro871Leu)	rs799917	Het	Benign
				*BRCA2*	c.1114A > C	p.(Asn372His)	rs144848	Het	Benign
					c.7397T > C	p.(Val2466Ala)	rs169547	Hom	Benign
					**c.7940T > C**	**p.(Leu2647Pro)**	**rs80359021**	**Het**	**Pathogenic**
P8	F	76	Pancreas	*BRCA1*	c.4900A > G	p.(Ser1634Gly)	rs1799966	Het	Benign
					c.3548A > G	p.(Lys1183Arg)	rs16942	Het	Benign
					c.3113A > G	p.(Glu1038Gly)	rs16941	Het	Benign
					c.2612C > T	p.(Pro871Leu)	rs799917	Het	Benign
				*BRCA2*	c.1114A > C	p.(Asn372His)	rs144848	Het	Benign
					c.1151C > T	p.(Ser384Phe)	rs41293475	Hom	Benign
					c.7397T > C	p.(Val2466Ala)	rs169547	Hom	Benign
P9	M	71	Pancreas	*BRCA1*	c.4900A > G	p.(Ser1634Gly)	rs1799966	Hom	Benign
					c.3548A > G	p.(Lys1183Arg)	rs16942	Hom	Benign
					c.3113A > G	p.(Glu1038Gly)	rs16941	Hom	Benign
					c.2612C > T	p.(Pro871Leu)	rs799917	Hom	Benign
					c.2077G > A	p.(Asp693Asn)	rs4986850	Het	Benign
				*BRCA2*	c.1114A > C	p.(Asn372His)	rs144848	Het	Benign
					c.7397T > C	p.(Val2466Ala)	rs169547	Hom	Benign
				*PALB2*	c.1676A > G	p.(Gln559Arg)	rs152451	Het	Benign
P10	M	71	Prostate	*BRCA1*	c.4900A > G	p.(Ser1634Gly)	rs1799966	Het	Benign
					c.3548A > G	p.(Lys1183Arg)	rs16942	Het	Benign
					c.3113A > G	p.(Glu1038Gly)	rs16941	Het	Benign
					c.2612C > T	p.(Pro871Leu)	rs799917	Het	Benign
				*BRCA2*	c.7397T > C	p.(Val2466Ala)	rs169547	Hom	Benign
P11	M	70	Pancreas	*BRCA1*	c.4054G > A	p.(Glu1352Lys)	rs80357202	Het	Benign
				*BRCA2*	c.1114A > C	p.(Asn372His)	rs144848	Het	Benign
					c.7397T > C	p.(Val2466Ala)	rs169547	Hom	Benign
P12	M	69	Pancreas	*BRCA2*	c.1114A > C	p.(Asn372His)	rs144848	Het	Benign
					c.7397T > C	p.(Val2466Ala)	rs169547	Hom	Benign
				*PALB2*	c.2014G > C	p.(Glu672Gln)	rs45532440	Het	Benign
					c.1676A > G	p.(Gln559Arg)	rs152451	Het	Benign
P13	M	61	Pancreas	*BRCA2*	c.7397T > C	p.(Val2466Ala)	rs169547	Hom	Benign
P14	F	58	Pancreas	*BRCA1*	c.4900A > G	p.(Ser1634Gly)	rs1799966	Het	Benign
					c.3548A > G	p.(Lys1183Arg)	rs16942	Het	Benign
					c.3113A > G	p.(Glu1038Gly)	rs16941	Het	Benign
					c.2612C > T	p.(Pro871Leu)	rs799917	Het	Benign
				*BRCA2*	c.865A > C	p.(Asn289His)	rs766173	Het	Benign
					c.2971A > G	p.(Asn991Asp)	rs1799944	Het	Benign
					c.7397T > C	p.(Val2466Ala)	rs169547	Hom	Benign
P15	F	58	Pancreas	*BRCA1*	c.4900A > G	p.(Ser1634Gly)	rs1799966	Hom	Benign
					c.3548A > G	p.(Lys1183Arg)	rs16942	Hom	Benign
					c.3113A > G	p.(Glu1038Gly)	rs16941	Hom	Benign
					c.2612C > T	p.(Pro871Leu)	rs799917	Hom	Benign
					c.2077G > A	p.(Asp693Asn)	rs4986850	Het	Benign
				*BRCA2*	c.7397T > C	p.(Val2466Ala)	rs169547	Hom	Benign
				*PALB2*	c.2014G > C	p.(Glu672Gln)	rs45532440	Het	Benign
					c.1676A > G	p.Gln559Arg	rs152451	Het	Benign
P16	F	80	Pancreas	*BRCA2*	c.7397T > C	p.(Val2466Ala)	rs169547	Hom	Benign
				*PALB2*	c.1676A > G	p.(Gln559Arg)	rs152451	Het	Benign
P17	M	75	Pancreas	*BRCA1*	c.4900A > G	p.(Ser1634Gly)	rs1799966	Het	Benign
					c.3548A > G	p.(Lys1183Arg)	rs16942	Het	Benign
					c.3113A > G	p.(Glu1038Gly)	rs16941	Het	Benign
					c.2612C > T	p.(Pro871Leu)	rs799917	Het	Benign
				*BRCA2*	c.1114A > C	p.(Asn372His)	rs144848	Hom	Benign
					c.7397T > C	p.(Val2466Ala)	rs169547	Hom	Benign
P18	M	57	Pancreas	*BRCA1*	c.4900A > G	p.(Ser1634Gly)	rs1799966	Het	Benign
					c.3548A > G	p.(Lys1183Arg)	rs16942	Het	Benign
					c.3113A > G	p.(Glu1038Gly)	rs16941	Het	Benign
					c.2612C > T	p.(Pro871Leu)	rs799917	Het	Benign
				*BRCA2*	c.7397T > C	p.(Val2466Ala)	rs169547	Hom	Benign
P19	F	70	Pancreas	*BRCA1*	c.4900A > G	p.(Ser1634Gly)	rs1799966	Het	Benign
					c.3548A > G	p.(Lys1183Arg)	rs16942	Het	Benign
					c.3113A > G	p.(Glu1038Gly)	rs16941	Het	Benign
					c.2612C > T	p.(Pro871Leu)	rs799917	Het	Benign
					c.2077G > A	p.(Asp693Asn)	rs4986850	Het	Benign
				*BRCA2*	c.7397T > C	p.(Val2466Ala)	rs169547	Hom	Benign
P20	M	78	Pancreas	*BRCA1*	c.4900A > G	p.(Ser1634Gly)	rs1799966	Hom	Benign
					c.3548A > G	p.(Lys1183Arg)	rs16942	Hom	Benign
					c.3113A > G	p.(Glu1038Gly)	rs16941	Hom	Benign
					c.2612C > T	p.(Pro871Leu)	rs799917	Hom	Benign
				*BRCA2*	c.7397T > C	p.(Val2466Ala)	rs169547	Hom	Benign
				*PALB2*	c.3451C > T	p.(Leu1151Phe)	rs786203462	Het	UCV
					c.1676A > G	p.(Gln559Arg)	rs152451	Het	Benign
P21	M	60	Prostate	*BRCA2*	c.7397T > C	p.(Val2466Ala)	rs169547	Hom	Benign
P22	F	75	Pancreas	*BRCA2*	c.7057G > C	p.(Gly2353Arg)	rs80358935	Het	UCV
					c.7397T > C	p.(Val2466Ala)	rs169547	Hom	Benign
				*PALB2*	c.2993G > A	p.(Gly998Glu)	rs45551636	Het	Benign
					c.2014G > C	p.(Glu672Gln)	rs45532440	Het	Benign
					c.1676A > G	p.(Gln559Arg)	rs152451	Het	Benign
P23	M	28	Pancreas	*BRCA1*	c.5019G > A	p.(Met1673Ile)	rs1799967	Het	Benign
					c.4900A > G	p.(Ser1634Gly)	rs1799966	Het	Benign
					c.3548A > G	p.(Lys1183Arg)	rs16942	Het	Benign
					c.3113A > G	p.(Glu1038Gly)	rs16941	Het	Benign
					c.2612C > T	p.(Pro871Leu)	rs799917	Het	Benign
				*BRCA2*	c.1114A > C	p.(Asn372His)	rs144848	Hom	Benign
					c.7397T > C	p.(Val2466Ala)	rs169547	Hom	Benign
P24	F	70	Pancreas	*BRCA1*	c.3119G > A	p.(Ser1040Asn)	rs4986852	Het	Benign
					c.1067A > G	p.(Gln356Arg)	rs1799950	Het	Benign
				*BRCA2*	c.865A > C	p.(Asn289His)	rs766173	Het	Benign
					c.2971A > G	p.(Asn991Asp)	rs1799944	Het	Benign
					c.7397T > C	p.(Val2466Ala)	rs169547	Hom	Benign
P25	M	70	Pancreas	*BRCA1*	c.4900A > G	p.(Ser1634Gly)	rs1799966	Het	Benign
					c.3548A > G	p.(Lys1183Arg)	rs16942	Het	Benign
					c.3113A > G	p.(Glu1038Gly)	rs16941	Het	Benign
					c.2612C > T	p.(Pro871Leu)	rs799917	Het	Benign
					c.1067A > G	p.(Gln356Arg)	rs1799950	Het	Benign
				*BRCA2*	c.1114A > C	p.(Asn372His)	rs144848	Het	Benign
					c.7397T > C	p.(Val2466Ala)	rs169547	Hom	Benign
				*PALB2*	c.1676A > G	p.(Gln559Arg)	rs152451	Het	Benign
P26	M	51	Pancreas	*BRCA2*	c.1114A > C	p.(Asn372His)	rs144848	Hom	Benign
					c.7397T > C	p.(Val2466Ala)	rs169547	Hom	Benign
P27	F	43	Breast	*BRCA1*	c.4900A > G	p.(Ser1634Gly)	rs1799966	Het	Benign
					c.3548A > G	p.(Lys1183Arg)	rs16942	Het	Benign
					c.3113A > G	p.(Glu1038Gly)	rs16941	Het	Benign
					c.2612C > T	p.(Pro871Leu)	rs799917	Het	Benign
					c.1067A > G	p.(Gln356Arg)	rs1799950	Het	Benign
				*BRCA2*	c.1114A > C	p.(Asn372His)	rs144848	Het	Benign
					**c.6468_6469delTC**	**p.(Gln2157IlefsTer18)**	**rs80359596**	**Het**	**Pathogenic**
					c.7397T > C	p.(Val2466Ala)	rs169547	Hom	Benign
P28	F	71	Breast	*BRCA1*	c.3548A > G	p.(Lys1183Arg)	rs16942	Het	Benign
					c.3113A > G	p.(Glu1038Gly)	rs16941	Het	Benign
					c.2612C > T	p.(Pro871Leu)	rs799917	Het	Benign
					c.2077G > A	p.(Asp693Asn)	rs4986850	Het	Benign
				*BRCA2*	c.7397T > C	p.(Val2466Ala)	rs169547	Hom	Benign
P29	F	65	Breast	*BRCA1*	c.4900A > G	p.(Ser1634Gly)	rs1799966	Het	Benign
					c.3548A > G	p.(Lys1183Arg)	rs16942	Het	Benign
					c.3113A > G	p.(Glu1038Gly)	rs16941	Het	Benign
					c.2612C > T	p.(Pro871Leu)	rs799917	Het	Benign
					c.457A > T	p.(Ser153Cys)	n.r.	Het	UCV
				*BRCA2*	c.865A > C	p.(Asn289His)	rs766173	Hom	Benign
					c.2971A > G	p.(Asn991Asp)	rs1799944	Hom	Benign
					c.7397T > C	p.(Val2466Ala)	rs169547	Hom	Benign
P30	F	70	Breast	*BRCA2*	**c.5796_5797delTA**	**p.(His1932GInfsTer12)**	**rs80359537**	**Het**	**Pathogenic**
					c.7397T > C	p.(Val2466Ala)	rs169547	Hom	Benign
P31	F	36	Breast	*BRCA1*	c.4900A > G	p.(Ser1634Gly)	rs1799966	Het	Benign
					c.3548A > G	p.(Lys1183Arg)	rs16942	Het	Benign
					c.3113A > G	p.(Glu1038Gly)	rs16941	Het	Benign
					c.2612C > T	p.(Pro871Leu)	rs799917	Het	Benign
				*BRCA2*	c.7397T > C	p.(Val2466Ala)	rs169547	Hom	Benign
P32	F	33	Breast	*BRCA1*	c.4900A > G	p.(Ser1634Gly)	rs1799966	Het	Benign
					c.3548A > G	p.(Lys1183Arg)	rs16942	Het	Benign
					c.3113A > G	p.(Glu1038Gly)	rs16941	Het	Benign
					c.2612C > T	p.(Pro871Leu)	rs799917	Het	Benign
					c.1067A > G	p.(Gln356Arg)	rs1799950	Het	Benign
				*BRCA2*	c.1114A > C	p.(Asn372His)	rs144848	Het	Benign
					c.5744C > T	p.(Thr1915Met)	rs4987117	Het	Benign
					c.7397T > C	p.(Val2466Ala)	rs169547	Hom	Benign
P33	F	34	Breast	*BRCA1*	c.2612C > T	p.(Pro871Leu)	rs799917	Het	Benign
				*BRCA2*	c.7397T > C	p.(Val2466Ala)	rs169547	Hom	Benign
P34	F	35	Breast	*BRCA1*	c.4900A > G	p.(Ser1634Gly)	rs1799966	Hom	Benign
					c.3548A > G	p.(Lys1183Arg)	rs16942	Hom	Benign
					c.3113A > G	p.(Glu1038Gly)	rs16941	Hom	Benign
					c.2612C > T	p.(Pro871Leu)	rs799917	Hom	Benign
				*BRCA2*	c.1114A > C	p.(Asn372His)	rs144848	Het	Benign
					c.7397T > C	p.(Val2466Ala)	rs169547	Hom	Benign
P35	F	39	Breast	*BRCA1*	c.4900A > G	p.(Ser1634Gly)	rs1799966	Hom	Benign
					c.3548A > G	p.(Lys1183Arg)	rs16942	Hom	Benign
					c.3113A > G	p.(Glu1038Gly)	rs16941	Hom	Benign
					c.2612C > T	p.(Pro871Leu)	rs799917	Hom	Benign
					c.2077G > A	p.(Asp693Asn)	rs4986850	Het	Benign
				*BRCA2*	c.1114A > C	p.(Asn372His)	rs144848	Het	Benign
					c.7397T > C	p.(Val2466Ala)	rs169547	Hom	Benign
P36	F	41	Breast	*BRCA1*	c.3119G > A	p.(Ser1040Asn)	rs4986852	Het	Benign
				*BRCA2*	c.1114A > C	p.(Asn372His)	rs144848	Het	Benign
					c.7397T > C	p.(Val2466Ala)	rs169547	Hom	Benign
P37	F	39	Breast	*BRCA1*	c.4900A > G	p.(Ser1634Gly)	rs1799966	Het	Benign
					c.3548A > G	p.(Lys1183Arg)	rs16942	Het	Benign
					c.3113A > G	p.(Glu1038Gly)	rs16941	Het	Benign
					c.2612C > T	p.(Pro871Leu)	rs799917	Het	Benign
				*BRCA2*	c.1114A > C	p.(Asn372His)	rs144848	Het	Benign
					c.7397T > C	p.(Val2466Ala)	rs169547	Hom	Benign
P38	F	38	Breast	*BRCA1*	c.4900A > G	p.(Ser1634Gly)	rs1799966	Het	Benign
					c.3548A > G	p.(Lys1183Arg)	rs16942	Het	Benign
					c.3113A > G	p.(Glu1038Gly)	rs16941	Het	Benign
					c.2612C > T	p.(Pro871Leu)	rs799917	Het	Benign
				*BRCA2*	c.7397T > C	p.(Val2466Ala)	rs169547	Hom	Benign
P39	F	27	Breast	*BRCA1*	c.4900A > G	p.(Ser1634Gly)	rs1799966	Het	Benign
					c.3548A > G	p.(Lys1183Arg)	rs16942	Het	Benign
					c.3113A > G	p.(Glu1038Gly)	rs16941	Het	Benign
					c.2612C > T	p.(Pro871Leu)	rs799917	Het	Benign
				*BRCA2*	c.7397T > C	p.(Val2466Ala)	rs169547	Hom	Benign
P40	F	29	Breast	*BRCA1*	c.4900A > G	p.(Ser1634Gly)	rs1799966	Het	Benign
					c.3548A > G	p.(Lys1183Arg)	rs16942	Het	Benign
					c.3113A > G	p.(Glu1038Gly)	rs16941	Het	Benign
					c.2612C > T	p.(Pro871Leu)	rs799917	Het	Benign
					c.2077G > A	p.(Asp693Asn)	rs4986850	Het	Benign
				*BRCA2*	c.1114A > C	p.(Asn372His)	rs144848	Hom	Benign
					c.7397T > C	p.(Val2466Ala)	rs169547	Hom	Benign
				*PALB2*	c.2993G > A	p.(Gly998Glu)	rs45551636	Het	Benign
					c.2014G > C	p.(Glu672Gln)	rs45532440	Het	Benign
					c.1676A > G	p.(Gln559Arg)	rs152451	Het	Benign
P41	F	39	Breast	*BRCA1*	c.3119G > A	p.(Ser1040Asn)	rs4986852	Het	Benign
				*BRCA2*	c.7397T > C	p.(Val2466Ala)	rs169547	Hom	Benign
P42	F	38	Breast	*BRCA1*	c.4900A > G	p.(Ser1634Gly)	rs1799966	Het	Benign
					c.3548A > G	p.(Lys1183Arg)	rs16942	Het	Benign
					c.3113A > G	p.(Glu1038Gly)	rs16941	Het	Benign
					c.2612C > T	p.(Pro871Leu)	rs799917	Het	Benign
				*BRCA2*	c.1114A > C	p.(Asn372His)	rs144848	Hom	Benign
					c.7397T > C	p.(Val2466Ala)	rs169547	Hom	Benign
				*PALB2*	c.1676A > G	p.(Gln559Arg)	rs152451	Het	Benign
P43	F	38	Breast	*BRCA2*	c.7397T > C	p.(Val2466Ala)	rs169547	Hom	Benign
P44	F	39	Breast	*BRCA1*	c.4900A > G	p.(Ser1634Gly)	rs1799966	Hom	Benign
					c.3548A > G	p.(Lys1183Arg)	rs16942	Hom	Benign
					c.3113A > G	p.(Glu1038Gly)	rs16941	Hom	Benign
					c.2612C > T	p.(Pro871Leu)	rs799917	Hom	Benign
					c.2077G > A	p.(Asp693Asn)	rs4986850	Het	Benign
				*BRCA2*	c.1114A > C	p.(Asn372His)	rs144848	Het	Benign
					c.7397T > C	p.(Val2466Ala)	rs169547	Hom	Benign
P45	F	36	Breast	*BRCA2*	c.1114A > C	p.(Asn372His)	rs144848	Het	Benign
					c.7397T > C	p.(Val2466Ala)	rs169547	Hom	Benign
P46	F	43	Breast	*BRCA1*	c.1067A > G	p.(Gln356Arg)	rs1799950	Het	Benign
				*BRCA2*	c.7397T > C	p.(Val2466Ala)	rs169547	Hom	Benign
P47	F	42	Breast	*BRCA2*	c.1114A > C	p.(Asn372His)	rs144848	Het	Benign
					c.5508T > G	p.(Asn1836Lys)	rs80358774	Het	Benign
					c.7397T > C	p.(Val2466Ala)	rs169547	Hom	Benign
				*PALB2*	c.2993G > A	p.(Gly998Glu)	rs45551636	Het	Benign
					c.2014G > C	p.(Glu672Gln)	rs45532440	Het	Benign
					c.1676A > G	p.(Gln559Arg)	rs152451	Het	Benign
P48	F	74	Breast	*BRCA1*	c.5019G > A	p.(Met1673Ile)	rs1799967	Het	Benign
					c.4900A > G	p.(Ser1634Gly)	rs1799966	Het	Benign
					c.3548A > G	p.(Lys1183Arg)	rs16942	Het	Benign
					c.3113A > G	p.(Glu1038Gly)	rs16941	Het	Benign
					c.2612C > T	p.(Pro871Leu)	rs799917	Het	Benign
				*BRCA2*	c.1114A > C	p.(Asn372His)	rs144848	Het	Benign
					c.7397T > C	p.(Val2466Ala)	rs169547	Hom	Benign

* According to Human Genome Variation Society (HGVS) guidelines; ID: identifier; y: years; M: male; F: female; Het: heterozygous; Hom: homozygous.

**Table 3:** Full list of copy number variants identified in each analyzed patient based on bioinformatic software prediction. Variants confirmed after MLPA validation are reported in bold.

Sample ID	Gender	Age at Diagnosis (y)	Kind of Cancer	Gene	Chr Position	Kind of CNV	Confirmed by MLPA
P3	M	66	Pancreas	*CHEK2*	Chr22: 29091641-29091919	ex12Del	No
P12	M	69	Pancreas	*BRCA1*	Chr17: 41249247-41251890	ex8-9Del	No
P28	F	71	Breast	***BRCA1***	**Chr17: 41219572-41223313**	**ex16-17Del**	**Yes**
P29	F	65	Breast	***BRCA1***	**Chr17: 41275950-41277259**	**ex2Dup**	**Yes**
P48	F	74	Breast	*BRCA2*	Chr13: 32906693-32906915	ex10Del	No

ID: identifier; y: years; Chr: chromosome; CNV: copy number variant; MLPA: multiplex ligation probe amplification; M: male; F: female; ex: exon; Del: deletion, Dup: duplication.

## Data Availability

Not applicable.

## Abbreviations

*BRCA1*, breast cancer susceptibility gene 1; *BRCA2*, breast cancer susceptibility gene 2; HBOC, hereditary breast and ovarian cancer syndrome; PARP, poly(ADP-ribose) polymerase; NGS, next generation sequencing; VUS, variant of uncertain significance; *CHEK2*, checkpoint kinase 2; *PALB2*, partner and localizer of BRCA2; NCCN, National Comprehensive Cancer Network; CNV, copy number variant; SNP, single-nucleotide variant; MLPA, multiplex ligation probe amplification; DIN, DNA integrity number; ID, identifier; Y, year; M, male; F, female; Het, heterozygous; Hom, homozygous; Chr, chromosome; Ex, exon; Del, deletion; Dup, duplication.
